# Endometriosis-derived iPSCs reveal conserved stromal maturation and endocrine responsiveness

**DOI:** 10.1126/sciadv.aeg2362

**Published:** 2026-08-19

**Authors:** Hannah McDowell, Shiyang Sun, Ross McNally, Cassandra Huerta, Huma Asif, Julia Yoon, Angel Alvarez, Sule Yildiz, K. Grace Foley, Christina Boots, Magdy Milad, J. Julie Kim

**Affiliations:** ^1^ Division of Reproductive Science in Medicine, Northwestern University, Chicago, IL, USA.; ^2^ Department of Pathology, University of Pittsburgh School of Medicine (UPSOM), Pittsburgh, PA, USA.; ^3^ Department of Neurology, Stem Cell Core, Northwestern University, Chicago, IL, USA.; ^4^ Division of Reproductive Endocrinology and Infertility, Northwestern University, Chicago, IL, USA.; ^5^ Division of Minimally Invasive Gynecologic Surgery, Department of Obstetrics and Gynecology, Northwestern University, Chicago, IL, USA.

## Abstract

Endometriosis is a chronic, hormone-dependent disease characterized by altered endometrial stromal function, but mechanistic and translational studies have been hindered by the lack of tractable human models. Here, we establish an induced pluripotent stem cell (iPSC)–based platform derived from patients with endometriosis to model endometrial stromal differentiation in a controlled human context. Using a defined differentiation protocol, endometriosis-derived iPSCs transition from pluripotency through mesenchymal commitment toward stromal-like states and acquire transcriptional hormone responsiveness. Transcriptomic analyses reveal coordinated repression of pluripotency and proliferative programs with induction of stromal lineage signatures. Comparison with independent transcriptomic datasets demonstrated that in vitro–derived stromal cells progressively acquired gene expression profiles resembling eutopic endometrial stromal programs in endometriosis. Conditioned media from iPSC-derived stromal cells also induced transcriptional reprogramming in THP-1 macrophage-like cells. Together, these findings establish a patient-derived platform for investigating stromal differentiation and stromal-immune interactions in endometriosis.

## INTRODUCTION

Endometriosis is a chronic gynecologic disease in which endometrial-like tissue establishes and persists outside of the uterus, most commonly within the peritoneal cavity and ovaries. The disease affects ∼10% of reproductive-aged females and is often associated with severe pelvic pain, infertility, and diminished quality of life (*1*). Despite its prevalence, the etiologies and mechanisms underlying endometriosis remain incompletely understood, limiting the development of effective diagnostic and therapeutic strategies. Increasing evidence implicates endometrial stromal dysfunction as a central contributor to disease pathophysiology, influencing aberrant hormone responsiveness, inflammatory signaling, and tissue remodeling within both eutopic and ectopic environments (*2*–*5*).

Studies of primary human endometrial stromal cells from endometriosis are constrained by limited tissue availability, variability in donor cycle stage, and the finite life span of stromal cells, as they undergo phenotypic changes after limited passages in culture, while animal models do not fully recapitulate human endometrial biology or disease heterogeneity (*6*, *7*). Moreover, most in vitro systems capture static endpoints rather than the dynamic process of stromal differentiation and endocrine response. Human-induced pluripotent stem cells (iPSCs) offer a powerful tool for studying normal physiology and disease. Takahashi and Yamanaka (*8*) made the seminal discovery that mouse skin fibroblasts could be reprogrammed into a pluripotent state using a cocktail of pluripotency transcription factors Oct4, Sox2, Klf4, and c-Myc. These iPSCs are similar to embryonic cells in terms of morphology, gene expression, differentiation status, and epigenetic pattern, both in culture and in vivo (*9*). Hence, iPSCs have provided the scientific community with a powerful platform to study conditions and diseases in which primary tissue or cells are difficult to obtain, such as tissues of the reproductive tract. iPSC application to endometrial biology has been minimal. In particular, no studies to date have systematically derived, differentiated, and transcriptionally characterized iPSCs generated from patients with endometriosis. This gap is notable given the potential of iPSC-based systems to enable reproducible, scalable, and patient-specific interrogation of endometrial cell states that are otherwise difficult to study longitudinally in humans.

Here, we establish a human iPSC-based model of endometrial stromal differentiation derived from patients with endometriosis. Using a defined, stepwise differentiation protocol, we directed endometriosis-derived iPSCs through mesenchymal commitment toward stromal-like states and profiled transcriptional dynamics across differentiation and hormone exposure using RNA sequencing (RNA-seq). To provide biological context for these in vitro–derived cell states, we benchmarked their transcriptional profiles against an independent human stromal reference dataset generated from endometriosis patient tissues. This integrative approach enabled assessment of transcriptional convergence toward stromal programs.

Our results demonstrate that endometriosis-derived iPSCs undergo coordinated loss of pluripotency, acquisition of stromal lineage programs, and progressive emergence of canonical hormone-responsive transcriptional states. These changes occur reproducibly across independent patient lines (*n* = 7) and show convergence toward transcriptional states observed in eutopic endometrial stromal cells in endometriosis. Together, this work establishes a patient-derived platform for modeling endometrial stromal differentiation in a disease-relevant context and provides a foundation for future studies aimed at defining disease-specific mechanisms.

## RESULTS

### Endometriosis-derived iPSCs undergo stepwise differentiation toward stromal lineages

Peripheral blood mononuclear cells (PBMCs) were collected from females with clinically diagnosed endometriosis (*n* = 7), defined by the surgical confirmation of endometrial implants, adhesions, or ovarian cysts on one or both ovaries. The ages of patients ranged from 27 to 43 years with a mixed reported race. Clinical information is provided in table S1.

**Fig. 1.**
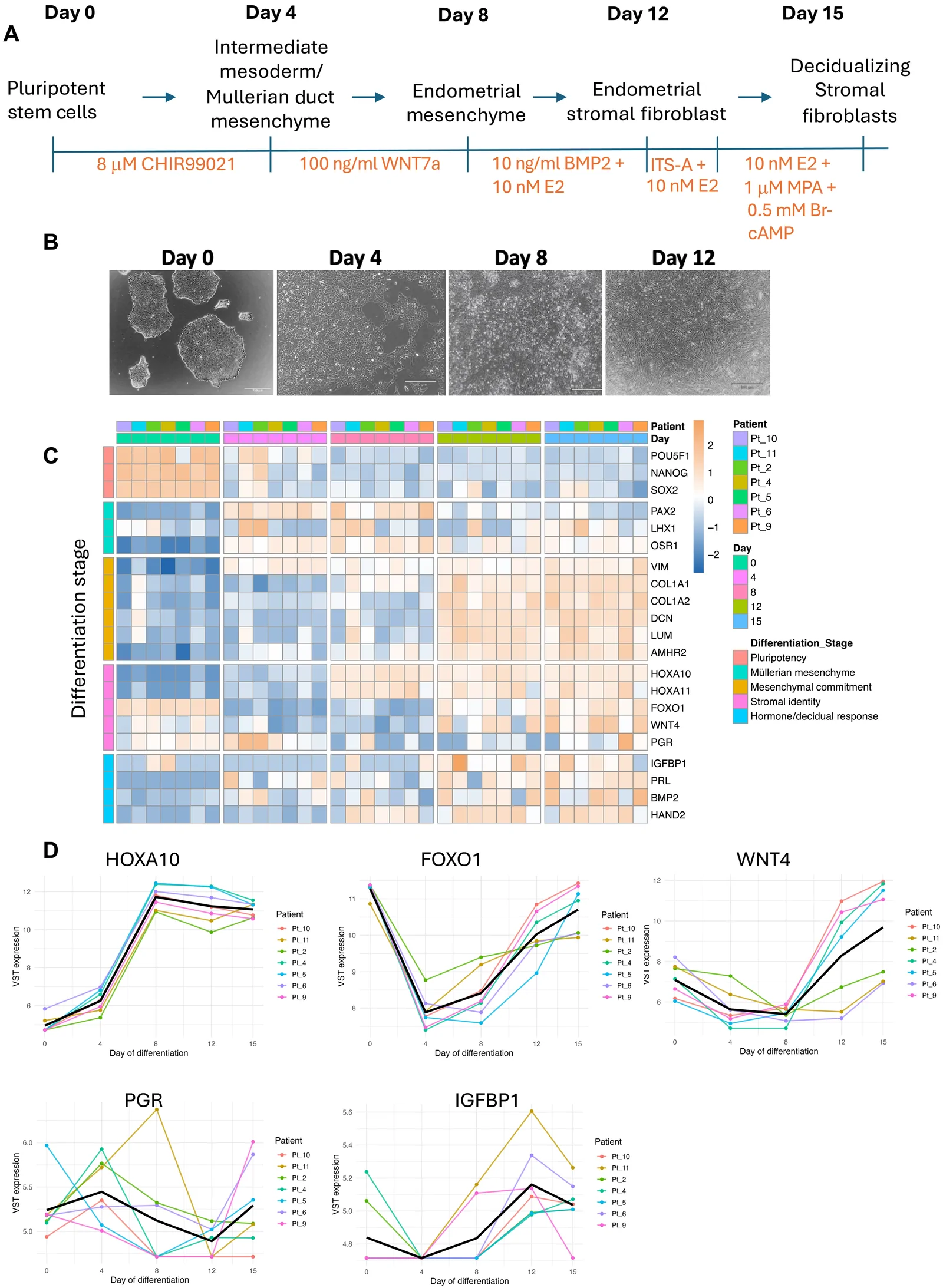
iPSCs derived from patients with endometriosis can be differentiated into endometrial stromal fibroblast-like cells EMSFs. (**A**) Differentiation protocol adapted from Cheung *et al.* (*10*) for the generation of endometrial stromal fibroblasts. (**B**) Representative images of cells at days 0, 4, 8, and 12 of differentiation. Scale bars, 750 μm. (**C**) Heatmap of curated marker genes across differentiation from days 0 to 15. Variance-stabilized expression values were centered and scaled by gene; samples were ordered by differentiation day. Columns represent samples and rows represent genes, highlighting coordinated, stage-dependent transcriptional programs across patient-derived lines. (**D**) Line plots showing variance-stabilized expression of selected canonical markers across time points. Each line represents an individual patient-derived iPSC line, with the dark black line representing the mean. VST, Variance Stabilizing Transformation.

PBMCs were reprogrammed to iPSCs, expanded, and stored. Differentiation of the iPSCs to endometrial mesenchymal stromal fibroblasts (EMSFs) followed the protocol by Cheung *et al.* (*10*) which differentiated iPSCs through the intermediate mesoderm, to Müllerian duct mesenchyme, and endometrial mesenchyme into EMSFs which could then be decidualized (Fig. 1A) (*10*). iPSCs from each patient were plated at a range of densities determined by the rate of expansion and space needed for growth and differentiation of each line throughout the 15-day protocol. The morphology of the iPSCs was monitored daily to ensure spontaneous differentiation did not occur (Fig. 1B), and RNA-seq was done using samples from five time points of differentiation (days 0, 4, 8, 12, and 15).

Global transcriptional analysis revealed a coordinated and progressive loss of pluripotency-associated gene expression during early differentiation. Visualization of stemness-associated gene expression across all patient-derived lines, including *POU5F1* (*OCT4*), *SOX2*, and *NANOG*, was robustly down-regulated with time and remained mostly suppressed throughout subsequent stages (Fig. 1C). This uniform loss of pluripotency programs across independent iPSC lines indicates that endometriosis-derived iPSCs reproducibly exit the pluripotent state under defined differentiation conditions. Concomitantly, expression of gene markers of Mullerian mesenchyme, including Paired Box-2 (*PAX2*,), LIM Homeobox 1 (*LHX1*), and Odd-Skipped Related (*OSR*) mesenchymal (*VIM*, *COL1A1*, *COL1A2*, *DCN*, *LUM*, and *AMHR2*), stromal-associated (*HOXA10*, *HOXA11*, *FOXO1*, *WNT4*, and *PGR*), and decidual genes (*IGFBP1*, *PRL*, and *BMP2*) emerged, defining a continuous transcriptional trajectory away from pluripotency toward a differentiated stromal state. The degree of variability in the time and degree of expression between the patient lines, regardless of in vitro cultures throughout the 15 days, is demonstrated in the heatmap, as well as the line graphs, shown for a subset of genes (Fig. 1D). Together, these data establish a robust and reproducible framework for directing patient-derived iPSCs toward stromal-like lineages and provide the foundation for subsequent analyses of stromal maturation and hormone responsiveness.

### Endometriosis-derived iPSCs undergo a coordinated transcriptional trajectory toward stromal maturation

**Fig. 2.**
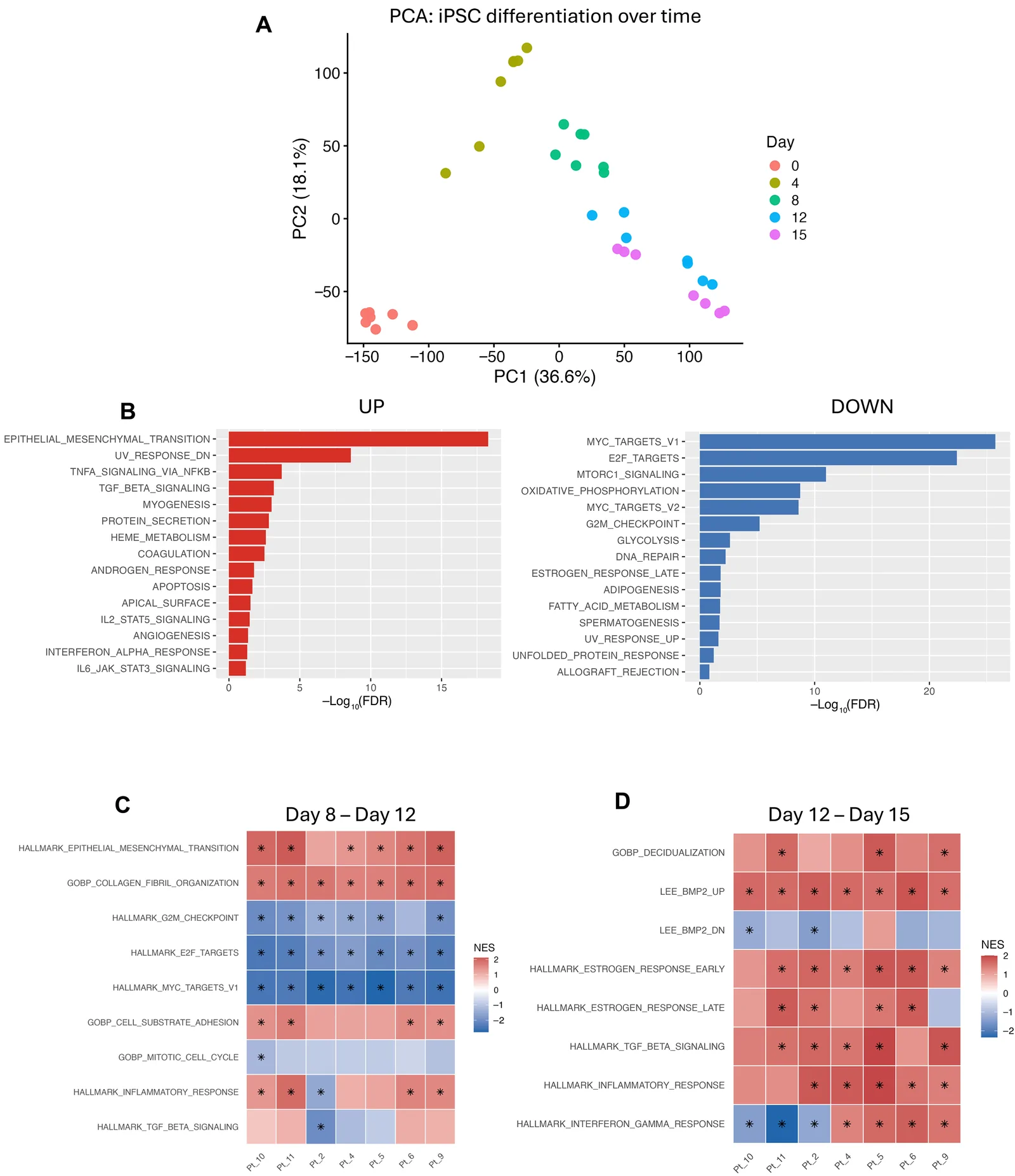
Trajectory-resolved transcriptional programs during stromal differentiation. (**A**) PCA of variance-stabilized gene expression showing separation of samples by differentiation day (days 0, 4, 8, 12, and 15). Each point represents an individual sample, colored by time point. (**B**) Pathway enrichment analysis across the full differentiation trajectory (days 0 to 15). Gene sets from the MSigDB [Hallmark (H) and Gene Ontology Biological Process] were evaluated using ranked genes associated with the differentiation trajectory. Bar plots show significantly enriched pathways (FDR < 0.05), with positive enrichment indicating pathways associated with genes increasing across differentiation (left, red) and negative enrichment indicating pathways associated with genes decreasing across (right, blue), plotted as −log_10_(FDR). (**C** and **D**) Heatmaps showing normalized enrichment scores (NES) from gene set enrichment analyses performed independently for each patient-derived line, using ranked gene lists from pairwise comparisons. Comparisons include day 8 versus day 12 (C) and day 12 versus day 15 following hormone exposure (D). Gene sets were obtained from MSigDB Hallmark collections. Columns represent individual patient-derived lines. Asterisks indicate positive False Discovery Rate (pFDR) < 0.05.

To define the global transcriptional trajectory of endometriosis-derived iPSCs during stromal differentiation, we performed principal components analysis (PCA) on RNA-seq profiles from patient iPSC lines across differentiation days 0 to 15 (Fig. 2A). Samples segregated primarily by differentiation stage along PC1, with early time points (days 0 to 4) clearly separated from later stages (days 8 to 15), indicating a dominant axis of transcriptional progression. Intermediate stages occupied distinct positions between pluripotent and stromal states, consistent with stepwise lineage commitment rather than abrupt state transitions. The day 12 and day 15 stages showed reduced separation, consistent with maintenance of stromal identity during the decidual hormone response.

Differential expression analysis revealed substantial transcriptional remodeling during stromal differentiation. Sequential comparisons identified 2863 differentially expressed genes (DEGs) between day 8 and day 12 and 2185 DEGs between day 12 and day 15 (adjusted *P* < 0.05). The DEGs between day 8 and day 12 were up- (1582 up) and down-regulated (1281 down). These findings align with the PCA (Fig. 2A), which demonstrates progressive separation across differentiation stages, with the most pronounced shift occurring at intermediate-to-late stages.

To identify biological programs associated with progression along the stromal differentiation trajectory, we performed pathway enrichment analysis on genes whose expression increased or decreased across the differentiation axis (Fig. 2B). Pathways enriched among genes increasing across differentiation included epithelial-mesenchymal transition, tumor necrosis factor–α signaling via nuclear factor κB, transforming growth factor–β (TGFβ) signaling, angiogenesis, and extracellular matrix–associated programs, consistent with acquisition of mesenchymal and stromal characteristics. In contrast, pathways enriched among genes decreasing across differentiation included cell cycle progression, MYC target gene expression, and proliferative signaling indicating coordinated repression of pluripotency-associated and proliferative programs during differentiation.

To further characterize stage-specific transcriptional changes, we performed gene set enrichment analysis (GSEA) using ranked gene lists derived from differential expression between time points for each patient-derived iPSC line. Gene sets were considered for downstream analysis if they achieved false discovery rate (FDR) *q* < 0.05 in at least one patient-derived line and demonstrated consistent directionality of enrichment across patients, with biological relevance to stromal maturation and hormone responsiveness. Comparison of day 8 and day 12 samples revealed enrichment of stromal maturation–associated pathways across patient lines, including extracellular matrix organization, epithelial-mesenchymal transition, and inflammatory response by day 12 (Fig. 2C, red). In contrast, pathways related to cell cycle progression and MYC target gene expression were enriched among genes ranked lower at day 12 (blue), consistent with repression of proliferative programs. Asterisks denote pathways meeting statistical significance after FDR correction (FDR < 0.05). These patterns were reproducible across individual patient lines, indicating a coordinated transcriptional transition toward stromal identity rather than patient-specific outlier effects. Variability was observed in TGFβ signaling across patient lines: Only one of seven exhibited a significant decrease in enrichment between days 8 and 12, while four showed upward trends and two showed downward trends.

Comparison of day 12 and day 15 samples following estradiol, progestin, and cyclic adenosine 3′,5′-monophosphate (cAMP) exposure using GSEA revealed induction of hormone-responsive transcriptional programs, including decidualization-associated pathways, TGFβ signaling, Bone Morphogenetic Protein 2 (BMP2) pathway, and estrogen response (early and late) (Fig. 2D). These patterns were reproducibly observed across individual patient lines, with the exception of interferon-γ response, which exhibited interline variability. This variability suggests intrinsic differences in regulatory capacity among endometriosis-derived lines despite shared differentiation trajectories.

### Endometriosis-relevant pathway alignment

**Fig. 3.**
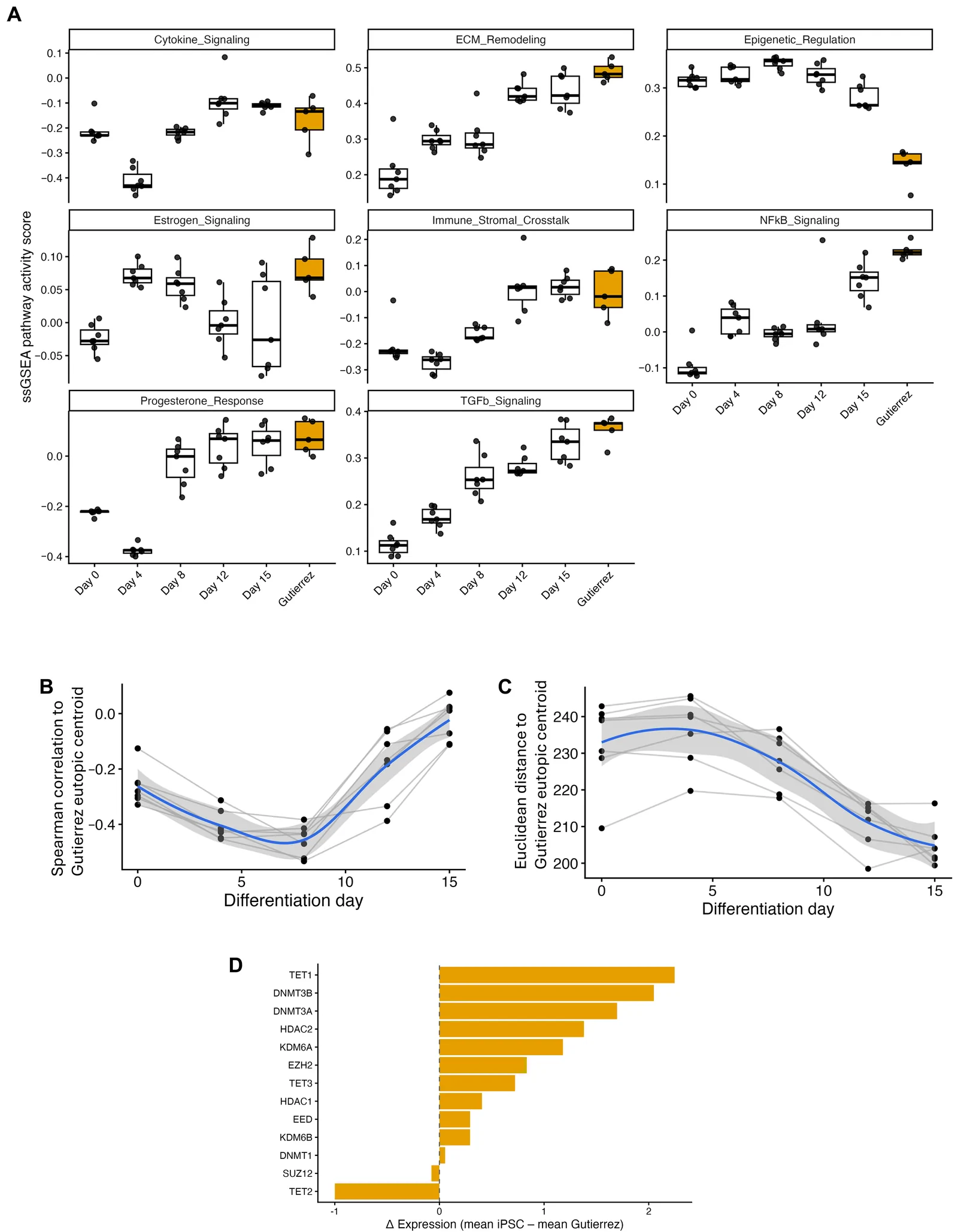
Progressive convergence of iPSC-derived stromal cells toward eutopic stromal programs of endometriosis. (**A**) Box plots of pathway activity scores derived from scores across differentiation (days 0, 4, 8, 12, and 15) and the adult eutopic stromal reference cohort from the Gutierrez study. ssGSEA was performed using GSVA, and each point represents an individual patient-derived line. The Gutierrez cohort comprises RNA-seq profiles of primary eutopic endometrial stromal cells from patients with endometriosis and is used here as a stromal reference. (**B**) Spearman correlation between each iPSC-derived sample and the centroid of the Gutierrez stromal cohort (C1 to C5) plotted across differentiation time. Gray lines indicate individual lines, and the blue line represents the smoothed average. Lower negative values indicate greater transcriptional divergence to the reference (**C**) Euclidean distance between each iPSC-derived sample and the Gutierrez stromal centroid across differentiation time. Lower values indicate greater transcriptional similarity to the reference. (**D**) Bar plot of differences in mean expression (Δ expression = mean iPSC − mean Gutierrez) for selected epigenetic regulator genes.

To evaluate disease-relevant stromal maturation, we used single-sample gene set enrichment analysis (ssGSEA) to quantify pathway activity, which generates per-sample enrichment scores based on ranked gene expression rather than differential testing. ssGSEA scores from iPSC-derived stromal cells were compared with those of the eutopic endometrial stromal cells from patients with endometriosis reported by Rodriguez Gutierrez *et al.* (*5*). Only the stromal signature genes were used, thus allowing assessment of how closely the iPSC-derived cells recapitulate disease-relevant stromal transcriptional programs. In the Gutierrez dataset, RNA-seq was performed on cultured stromal cells from eutopic endometrium of endometriosis and endometriotic lesions without hormonal treatment; here, we focused on the eutopic stromal cohort (*n* = 5) and compared it with iPSC-derived differentiation samples from days 0 to 15 (*n* = 7 lines). Box plots were used to compare pathway activity scores between iPSC-derived stromal cells and eutopic endometrial stromal cells from patients with endometriosis (*5*). For each pathway, ssGSEA scores were computed for individual samples, and distributions were compared across datasets using box plots (Fig. 3A). Early iPSC-derived cells (days 0 to 4) exhibited pathway activity distributions distinct from adult eutopic stroma, whereas later differentiation stages (days 12 to 15) demonstrated a marked shift toward the adult reference, reflected by increased overlap and narrowing of score distributions. To further quantify the differences and similarities of the iPSC-derived cells to disease-relevant eutopic endometrial stromal cells, we calculated the Spearman correlation (Fig. 3B) and Euclidean distance (Fig. 3C) using variance-stabilized expression values. Spearman correlation values increased from negative toward zero over the course of differentiation, indicating reduced discordance in gene ranking. In parallel, Euclidean distance decreased over time, reflecting increasing similarity in overall expression magnitude. By day 15, most lines showed their highest correlation and lowest distance relative to the Gutierrez eutopic stromal reference, suggesting that later-stage differentiated iPSC-derived cells more closely recapitulate the disease-relevant stromal identity.

In contrast, epigenetic regulatory programs remained distinct from adult eutopic stroma across all stages, with persistently elevated ssGSEA scores in iPSC-derived cells and limited convergence during differentiation. To investigate the molecular basis of this epigenetic pathway difference, we examined expression of core chromatin regulatory genes within this gene set. This analysis revealed coordinated up-regulation of multiple components of the DNA methylation and demethylation machinery in iPSC-derived stromal cells, including *TET1*, *DNMT3A*, and *DNMT3B*, each showing substantially higher mean expression compared with adult eutopic stromal samples (Fig. 3D). Additional chromatin-associated regulators showed the same directional shift, except for *TET2*, indicating that the elevated ssGSEA scores reflect a broad enhancement of epigenetic remodeling programs. Together, these findings indicate that while iPSC-derived stromal cells recapitulate key disease-relevant transcriptional programs observed in eutopic stroma from patients with endometriosis, they have a distinct epigenetic regulatory profile characterized by increased expression of chromatin remodeling machinery.

### Endometriosis stromal conditioned media reprograms macrophage transcriptional programs

**Fig. 4.**
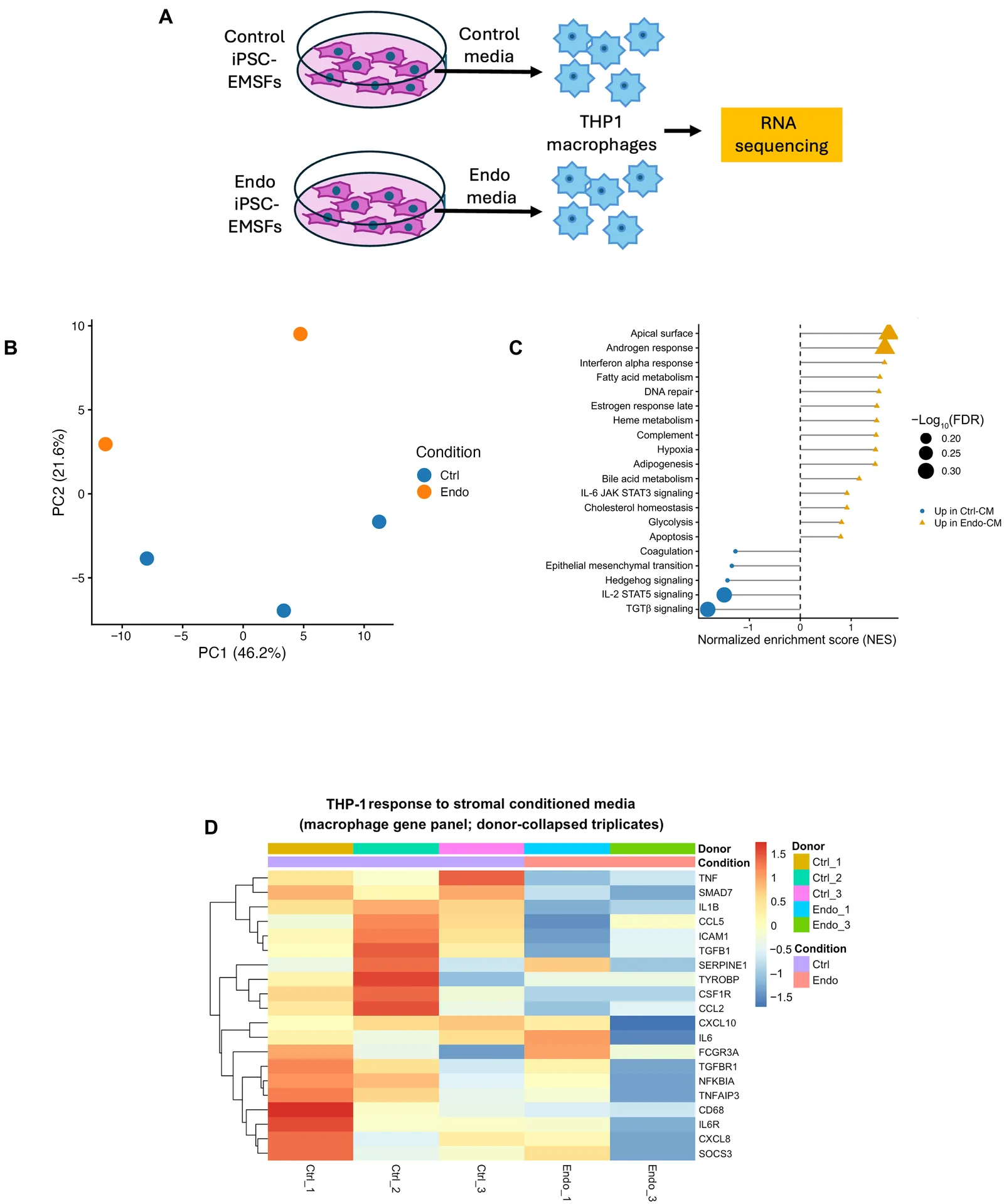
Endometriosis-derived stromal conditioned media induces transcriptional reprogramming in THP-1 macrophages. (**A**) Schematic of the experimental design. Conditioned media from control or endometriosis-derived iPSC stromal cultures was applied to phorbol 12-myristate 13-acetate–differentiated THP-1 macrophages, followed by RNA extraction and sequencing. (**B**) PCA of THP-1 transcriptomes after treatment with control CM (Ctrl-CM) or endometriosis CM (Endo-CM), showing separation by treatment condition. Each point represents a donor-collapsed sample. (**C**) Hallmark GSEA comparing Endo-CM versus Ctrl-CM–treated THP-1 cells. Pathways are plotted by NES, with point size indicating −log_10_(FDR) and direction indicating enrichment in Endo-CM or Ctrl-CM conditions. (**D**) Heatmap of a curated macrophage gene panel showing variance-stabilized expression values in THP-1 cells treated with Ctrl-CM or Endo-CM. Expression values were averaged across technical triplicates for each donor and visualized by hierarchical clustering, highlighting treatment-associated transcriptional changes (control *n* = 3; endometriosis *n* = 2). IL-6, interleukin-6; JAK, Janus kinase; signal transducer and activator of transcription 3.

Having established that iPSC-derived stromal cells progressively acquire a transcriptional state that resembles disease-relevant eutopic stroma, we next asked whether this state is accompanied by changes in stromal-derived signals capable of modulating immune cell programs. To address this, THP-1 cells were treated with conditioned media from day 12 iPSC-derived stromal cultures from endometriosis/adenomyosis (Endo-CM) or control stromal cultures (Ctrl-CM) (Fig. 4A), followed by RNA-seq analysis (see Discussion regarding samples used). PCA revealed separation between conditions along PC2, which accounted for 21% of the variance, while the primary source of variation (PC1) was driven by patient-specific differences. These findings suggest that, although interpatient variability is a contributor to overall transcriptional variation, treatment-associated effects are detectable in the transcriptome. Unbiased Hallmark GSEA further identified coordinated differences in pathway activity between Endo-CM– and Ctrl-CM–treated cells (Fig. 4B). In particular, exposure to Endo-CM was associated with relative enrichment of pathways related to epithelial surface programs, androgen and estrogen responses, lipid and sterol metabolism, adipogenesis, and complement signaling, patterns consistent with altered metabolic and hormone-responsive states in macrophage-like cells. In contrast, gene sets associated with MYC targets, phosphatidylinositol 3-kinase–AKT–mammalian target of rapamycin signaling, mitotic spindle, ultraviolet response, and DNA repair showed relative depletion under Endo-CM conditions and were instead enriched in Ctrl-CM–treated cells. Notably, immune and profibrotic signaling pathways, including interleukin-2–signal transducer and activator of transcription 5 and TGFβ signaling, as well as epithelial-mesenchymal transition, were among the most strongly shifted programs, suggesting that endometriosis stromal conditioned media may influence macrophage activation and differentiation-related states.

To further probe these transcriptional changes at the level of macrophage-associated genes, we examined a focused gene panel in THP-1 cells treated with Ctrl-CM or Endo-CM. Gene expression values were collapsed across technical triplicates for each donor and visualized by hierarchical clustering (Fig. 4C). Endo-CM treatment was associated with higher expression of genes linked to macrophage differentiation and signaling, including *CSF1R*, *TYROBP*, *CD68*, *FCGR3A*, and *TGFB1*, together with modulation of inflammatory mediators such as *IL6*, *CXCL8*, *CCL2*, and *CXCL10*. Conversely, several canonical proinflammatory and stress-response genes, including *TNF*, *IL1B*, and *NFKBIA*, showed relatively lower expression under Endo-CM conditions compared to controls. Hierarchical clustering of DEGs revealed separation of expression patterns by treatment condition. However, as this analysis was performed on a selected set of genes, it does not represent an unbiased assessment of global sample relationships. Nevertheless, given the limited sample size and known interpatient variability among iPSC-derived lines, these findings should be interpreted cautiously and warrant validation in larger cohorts with additional biological replicates. These findings are exploratory and hypothesis generating.

## DISCUSSION

Endometriosis remains a challenging disease to study ex vivo, in part because of the limited availability of physiologically relevant human model systems. Although primary tissues and animal models have provided important insights, they are constrained by issues of access, heterogeneity, and incomplete representation of human stromal differentiation, hormonal responsiveness, and immune interactions (*6*, *7*). These limitations have slowed efforts to connect disease genetics and cell biology to function, motivating the development of renewable, patient-specific experimental platforms. We established patient-derived iPSCs to model endometriosis-associated stromal differentiation and hormone responsiveness in a controlled, human-relevant system. By leveraging iPSC technology, we establish a renewable and patient-specific platform that enables systematic interrogation of stromal maturation and endocrine responses in the context of endometriosis, providing a new experimental framework to study disease-relevant cell states and their functional consequences. Genome-wide association and genetic studies have established a heritable component to endometriosis risk, yet the functional consequences of most associated variants remain poorly understood (*11*–*15*). Because iPSCs retain the donor’s genetic background, including risk-associated alleles, disease-relevant regulatory variation is carried through reprogramming and differentiation. This creates an opportunity to use patient-derived iPSCs as a platform to link genetic variation to molecular and cellular phenotypes in a controlled setting and to directly test how specific variants or loci influence stromal differentiation, hormone responsiveness, immune cross-talk, or other disease-relevant processes. Beyond the specific findings reported here, iPSC-based models offer several unique advantages for studying endometriosis. iPSCs provide a renewable, patient-specific resource that can be differentiated into multiple relevant cell types from the same genetic background, enabling systematic studies of cell-cell interactions and tissue cross-talk. iPSCs are also amenable to genetic manipulation, including targeted perturbation of candidate genes or pathways, and to scalable drug or perturbation screens. Together, these features position iPSC-derived systems as a powerful experimental bridge between human genetics, cell biology, and translational therapeutic discovery.

An important and anticipated question is why endometriosis-derived iPSCs were not directly compared to control iPSCs in this study. While inclusion of matched control iPSC-derived stromal cells would strengthen assessment of disease specificity, several factors limited the interpretability of such comparisons in the current dataset. We reprogrammed four control lines (*n* = 4); however, the relatively small number and heterogeneity of iPSC lines limited statistical power and robustness for detecting disease-associated effects. On the basis of general RNA-seq power considerations and the known variation in iPSC models (*16*, *17*), detecting cohort-wide differences with moderate effect sizes would generally require in the order of approximately 10 to 15 independent donor lines per group to achieve sufficient statistical power, especially for heterogeneous disease phenotypes. Furthermore, validation of observed differences and comprehensive molecular characterization would be necessary to conclude that endometriosis-derived iPSCs harbor intrinsic genetic determinants. Endometriosis is a complex condition shaped by both intrinsic cellular factors and prolonged exposure to inflammatory, hormonal, and environmental cues in vivo. These influences can drive epigenetic and microenvironment-dependent alterations that may not be fully recapitulated in early-stage iPSC-derived stromal cells generated under controlled in vitro conditions. Hence, this highlights the importance of developmental stage and environmental context in shaping disease biology. In the current study, our primary objective was to establish and characterize the differentiation of iPSCs derived from patients with endometriosis into stromal-like cells. We demonstrate that these cells undergo a robust and coordinated transcriptional progression toward stromal identity, consistent with established differentiation frameworks. Comparison to the Gutierrez dataset, consisting of primary eutopic endometrial stromal cells from patients with endometriosis, provides biological context by demonstrating convergence toward in vivo stromal transcriptional programs. Broader comparisons to bulk tissue and single-cell datasets may provide additional insight; however, differences in cellular composition and experimental conditions limit direct comparability. Future studies incorporating larger, well-matched cohorts of control and endometriosis-derived iPSC lines processed in parallel, as well as models that integrate inflammatory and hormonal conditioning or targeted genetic perturbations, will be essential to rigorously define disease-specific phenotypes. We therefore frame the current work as a foundational step establishing a patient-derived stromal differentiation model in a disease-relevant context, upon which more comprehensive mechanistic and comparative studies can be built.

Differentiation of control iPSCs into endometrial stromal cells has already been demonstrated in prior work (*10*, *18*). The observed differentiation trajectory is consistent with the stepwise stromal lineage progression described by Cheung *et al.* (*10*), including loss of pluripotency and acquisition of stromal and decidual gene expression programs. Our RNA-seq analysis extends these findings by demonstrating that this process occurs as a continuous, transcriptome-wide transition while also revealing patient-specific variability. Notably, the number of independent patient-derived iPSC lines analyzed here exceeds what is typically reported in many disease-focused iPSC studies, which often rely on small cohorts of two to four lines per condition due to the substantial time, cost, and technical complexity of reprogramming, differentiation, and deep molecular profiling (*16*, *19*–*21*). Although modest in size, the cohort (*n* = 7) captures interpatient variability and supports the reproducibility of the observed transcriptional and functional patterns.

Despite originating from patients with endometriosis, we demonstrated that iPSC-derived cells in this system differentiate into stromal-like cells and mounting transcriptional responses to hormonal cues, indicating that core aspects of stromal identity and endocrine responsiveness are preserved or reestablished during differentiation. This finding suggests that disease-associated alterations in endometriosis may not reflect a simple loss of stromal fate or hormone response capacity, but rather more subtle rewiring of regulatory, metabolic, inflammatory, epigenetic, or signaling programs. The ability to recover these fundamental properties in vitro provides a critical foundation for dissecting which components of the stromal program are conserved and which are altered in a disease context. The elevated expression of DNA methylation and chromatin regulatory factors in the iPSC-derived cells may reflect ongoing stabilization of cell identity following in vitro differentiation and is different from the mature adult stromal cells from endometriosis, which is well known to have dysregulated epigenetic mechanisms (*22*). This distinction can be used for future experimental exposure of iPSCs to conditions of endometriosis, such as inflammation as a cause of epigenetic dysregulation.

Progesterone resistance is an established phenotype in endometriosis, but the underlying mechanisms are still not entirely clear. If this phenotype arises from intrinsic cellular factors, iPSC-derived models could provide a valuable system to investigate these mechanisms. In the current study, several decidual genes increased in endometriosis-derived iPSC lines following treatment with E2, MPA, and cAMP for 2 days, indicating that these cells are capable of responding to hormonal stimuli. However, determining whether this response is reduced compared to nondiseased cells would require more rigorous analysis than a simple side-by-side comparison, particularly given the known heterogeneity of decidual responses in both iPSC-derived and adult endometrial stromal cells from diseased and nondiseased patients. It is also possible that progesterone resistance observed in adult endometriotic stromal cells reflects cumulative epigenetic and microenvironmental influences acquired over time in vivo, rather than purely intrinsic differences present at the iPSC-derived stage. Future studies incorporating well-matched control cohorts, along with models that include inflammatory and hormonal conditioning, will be necessary to rigorously assess disease-specific hormonal responsiveness.

*PGR* expression was heterogeneous across patient-derived lines and increased, in some cases, before the stage at which estradiol is typically produced. Because *PGR* also serves as a marker of stromal/Müllerian differentiation, this early increase may reflect progressive lineage specification rather than direct induction by estradiol alone. Consistent with this, developmental studies in the mouse uterus demonstrate that progesterone receptor (PR) expression is initiated in the neonate in the uterine epithelia by postnatal day 3 (P3) and in the stroma by P6 (*23*, *24*). These findings suggest that *PGR* expression during stromal differentiation is not strictly dependent on mature estrogen-driven regulation. In the context of iPSC-derived differentiation, early *PGR* induction likely reflects developmental programming of stromal identity rather than canonical adult endocrine dynamics.

An important limitation of the macrophage conditioned media experiments is that one of the patient-derived iPSC lines used was initially classified as endometriosis but was subsequently confirmed by pathology to represent adenomyosis. This unexpected finding underscores both the clinical and biological overlap between these two estrogen-dependent uterine disorders and highlights the inherent challenges of disease classification based on preoperative or provisional diagnoses. As a result, the macrophage experiments should be viewed as preliminary and interpreted with caution, particularly given the small number of lines. Nevertheless, the observation that stromal conditioned media from this disease-context iPSC line was sufficient to induce transcriptional changes in macrophage-like cells suggests that altered stromal-immune cross-talk may represent a shared or convergent feature of related uterine pathologies. From this perspective, these results provide proof of concept that patient-derived iPSC stromal models can be used to functionally interrogate immune modulation while also motivating future studies using larger, rigorously phenotyped cohorts to distinguish disease-specific versus shared inflammatory and remodeling programs across endometriosis and adenomyosis.

In summary, this study establishes a patient-derived iPSC-based platform to study stromal differentiation and hormone responsiveness in a disease-relevant context using a human, renewable, and genetically defined system. iPSC-derived stromal cells can capture aspects of stromal differentiation and hormone-responsive states while revealing coordinated effects of stromal secreted factors on macrophage-like cells. Although the current cohort size and study design limit conclusions about disease-specific differences, this framework provides a scalable and mechanistically tractable approach to studying how genetic background, differentiation state, and microenvironmental signals interact in a disease-relevant context. This platform lays the foundation for future studies incorporating larger cohorts, targeted genetic perturbations, and therapeutic screening.

## MATERIALS AND METHODS

### Human subjects and blood collections

Patients with surgically diagnosed endometriosis were identified in the clinic at Northwestern Medicine and Prentice Women’s Hospital (Chicago, Illinois). Informed consent was obtained from all patients following an Institutional Review Board–approved protocol (IRB protocol #STU00210779). Endometriosis was diagnosed on the basis of visual identification of lesions during surgery and biopsy confirmation showing endometrial glands and/or stroma. Females in the control cohort were identified as those with normal reproductive history, eumenorrheic and denied symptoms of dysmenorrhea, other chronic pelvic pain, and infertility. The lack of symptoms and normal ultrasound indicated no endometriosis or at least the lack of severe endometriosis disease. Blood was collected in sodium heparin tubes (Becton Dickinson, #367871) for analysis, and PBMCs were isolated typically within 2 hours of collection using SepMate PBMC isolation tubes according to the manufacturer’s instructions (STEMCELL Technologies, #85460).

### Reprogramming to iPSCs

Reprogramming was performed at Northwestern’s Stem Cell Core. PBMCs were expanded in StemSpan SFEM II media with an Erythroid Expansion supplement (STEMCELL Technologies, #09655). Following PBMC expansion, a nonintegrating Sendai viral-based approach (*25*) (Cytotune 2.0, Thermo Fisher Scientific, #A16517) was used to introduce the four “Yamanaka reprogramming factors,” OCT4, SOX2, KLF4, and MYC (*26*). Clonal iPSC-like colonies were selected, expanded on embryonic stem cell -qualified Matrigel (Corning, #354277) in mTeSR plus (STEMCELL Technologies, #100-0274), and characterized to pass Northwestern’s Stem Cell Core quality control standards. At least three colonies for each line were selected after meeting the criteria for morphology, growth, sterility, and marker expression. iPSCs were expanded and analyzed to ensure >90% of colonies were free of differentiated cells. iPSCs were expanded and frozen stocks were made.

### Differentiation of iPSC to EMSFs

iPSCs were differentiated to EMSFs using a previously published protocol (*10*). Briefly, iPSCs were thawed at 37°C and pelleted at 100*g* for 5 min. The supernatant was discarded, and the remaining pellet of iPSCs was resuspended in mTeSR+ with 10 μM Y27632 and plated onto Corning Matrigel -human Embryonic Stem Cell. Qualified Matrix-coated tissue culture plates. To passage, cells were dissociated using ReLeSR dissociation reagent (STEMCELL Technologies, #100-0484) and pelleted at 100*g* for 5 min. The supernatant was discarded, and the remaining iPSC pellet was resuspended in Advanced RPMI 1640 (Gibco, #12633012) and plated onto Corning Matrigel hESC-Qualified Matrix-coated tissue culture plates at various densities according to the growth rate of each patient-derived iPSC line.

After a 24-hour colony establishment period, cells were treated with 8 μM CHIR99021 (STEMCELL Technologies, #72054) in Advanced RPMI with 1% GlutaMAX (Gibco, #35050061) for 4 days (days 0 to 4). The medium was then replaced with Advanced RPMI containing recombinant human WNT7a (100 ng/ml; Thermo Fisher Scientific, #120-31) for an additional 4 days (days 4 to 8). Next, cells were cultured in Advanced RPMI supplemented with recombinant human BMP2 (10 ng/ml; R&D Systems, #355-bm) and 10 nM β-estradiol (Sigma-Aldrich, #E2758) from day 8 to day 12. The medium was then changed to Dulbecco’s modified Eagle’s medium (DMEM)/F12 (no phenol) (Gibco, #11039-021) with GlutaMAX, 10 nM β-estradiol, and ITS-A (insulin-transferrin-selenium-sodium pyruvate; Gibco, #51-300-044) for 24 hours. Media on cells were then changed to phenol red-free DMEM/F12 containing 2% charcoal-stripped fetal bovine serum (FBS), 0.5 mM 8-Br-cAMP (STEMCELL Technologies, #73604), 1 μM medroxyprogesterone acetate (Sigma-Aldrich, #M1629), and 10 nM β-estradiol for 2 days (days 13 to 15) to induce decidualization.

### Conditioned media collection and macrophage cultures

Control (SSL008, SSL012, and SSL014) and endometriosis (PT735) and adenomyosis (PT774) iPSC lines were differentiated to EMSFs using the protocol described above. Advanced RPMI 1640 supplemented with 1% GlutaMAX replaced differentiation media on day 13. Conditioned media was collected from wells with cells at a consistent range of confluency 48 hours after reaching the EMSF stage (days 13 to 15, no decidual hormones). Media was collected and centrifuged at 100*g* for 5 min before supernatant filtration through a 0.2-μm filter. Three milliliters from each line was then immediately frozen for later use. This media was then added to macrophages differentiated from THP-1 monocytes (Sigma-Aldrich).

THP-1 monocytes were grown separately to 90% confluency in RPMI supplemented with 10% FBS and 2 mM l-glutamine. When cells reached targeted confluency, THP-1 cells were differentiated into macrophages with 100 nM phorbol 12-myristate 13-acetate (Sigma-Aldrich, #P1585) for 24 hours. Cells were washed three times with phosphate-buffered saline and then treated with conditioned media from the day 12 EMSFs combined in an equal ratio of 1:1 with RPMI supplemented with 10% FBS and 2 mM l-glutamine for 48 hours after which RNA was harvested. Paired Student’s *t* test was used to determine statistical significance.

### RNA extraction and sequencing

RNA was isolated with the RNAeasy Plus Minikit (QIAGEN, #74104) according to the manufacturer’s protocol. RNA samples were quantified using a Qubit 2.0 Fluorometer (Life Technologies), and RNA integrity was checked using Agilent TapeStation 4200 (Agilent Technologies). RNA integrity numbers greater than or equal to 9 were used for library preparation using the NEBNext Ultra II RNA Library Prep Kit for Illumina following the manufacturer’s instructions (New England Biolabs, #E7770L). RNA-seq libraries were prepared and sequenced according to standard protocols at the NUSeq Core Facility. First-strand and second-strand cDNAs were then synthesized. cDNA fragments were end-repaired and adenylated at the 3′ ends, and universal adapters were ligated to cDNA fragments, followed by index addition and library enrichment by limited-cycle polymerase chain reaction (PCR). The sequencing libraries were validated on the Agilent TapeStation (Agilent Technologies) and quantified using a Qubit 2.0 Fluorometer, as well as by quantitative PCR. RNA-seq was performed by the Northwestern University NUSeq Core Facility on an Illumina HiSeq 4000 platform, generating paired-end 50-bp reads at an average depth of approximately 28 million reads per sample. For THP-1 cells, RNA was sequenced by Admera Health (South Plainfield, NJ) and generated an average of approximately 28 million reads per sample.

### RNA-seq data processing and statistical analysis

All samples included in this study were derived from endometriosis patient iPSC lines and processed within a single sequencing batch. As a result, no batch correction was applied across patient lines, and batch was not included as a variable in the differential expression design. This approach enabled assessment of transcriptional changes across differentiation within a consistent technical background. Raw sequencing reads were quantified at the transcript level using Salmon (index version 4) against a GRCh38 cDNA reference transcriptome. Transcript-level abundances were summarized to gene-level counts using the tximport package for downstream analyses. Gene identifiers were mapped from Ensembl gene IDs to gene symbols using the org.Hs.eg.db annotation package (Bioconductor). When multiple Ensembl IDs mapped to the same gene symbol, counts were aggregated at the gene level. Genes with low expression were filtered by removing those with fewer than 10 counts in at least 80% of samples. The remaining genes were used for downstream normalization and differential expression analysis. Normalization and variance stabilization were performed using DESeq2 to generate variance-stabilized expression matrices for visualization and downstream analyses. All analyses were conducted in R (version ≥4.0) using Bioconductor packages. Gene sets for pathway analyses were obtained from the Molecular Signatures Database (MSigDB, Broad Institute), including the Hallmark (H), curated (C2), and Gene Ontology biological process collections, as specified for each analysis.

### Gene expression visualization and marker analysis

Expression trajectories of canonical endometrial stromal and decidual markers (*HOXA10*, *FOXO1*, *PGR*, *WNT4*, and *IGFBP1*) were visualized using variance-stabilized counts derived from the DESeq2 workflow. For each gene, variance-stabilized expression values were plotted across differentiation time points for each patient-derived line, with trajectories displayed separately by line. Marker genes associated with each stage of differentiation were visualized using curated gene sets, and heatmaps were generated to assess coordinated expression patterns across differentiation. For visualization, gene expression values were centered and scaled across samples, and samples were ordered by differentiation day.

### Trajectory-resolved pathway analysis

To identify global transcriptional programs operating across differentiation, pathway enrichment analysis was performed using ranked gene expression changes spanning the full day 0 to day 15 trajectory. For each gene, a trajectory statistic was computed to reflect monotonic changes across differentiation time points, and genes were ranked accordingly. GSEA was performed using the fgsea R package on the ranked gene list, using curated pathway collections including MSigDB Hallmark (H) gene sets and Gene Ontology biological processes. This rank-based approach evaluates whether genes within predefined pathways are enriched at the top or bottom of the ranked list without applying an arbitrary significance threshold. Pathways with significant enrichment were identified on the basis of normalized enrichment scores (NES) and FDR, and enrichment directionality was interpreted as progressive induction or repression over the differentiation trajectory.

### Pathway activity and reference-based similarity analyses

Pathway-level activity during differentiation and in comparison to eutopic endometrial stromal cells [Rodriguez Gutierrez *et al.* (*5*)] was assessed using ssGSEA implemented in the GSVA R/Bioconductor package (version 2.2.1; RRID:SCR_021058) using the “ssgsea” method (*27*). Enrichment scores were calculated from variance-stabilized expression values using curated MSigDB gene sets. Score distributions across differentiation time points (days 0, 4, 8, 12, and 15) and the Gutierrez reference cohort were visualized using box plots. The Gutierrez dataset was selected as a reference because it represents primary eutopic endometrial stromal cells under decidualization conditions, closely matching the biological context of the iPSC-derived stromal cells analyzed in this study. Global transcriptomic similarity to the Gutierrez eutopic stromal reference was quantified using two complementary metrics: Spearman correlation and Euclidean distance to the reference centroid (C1 to C5), both computed from variance-stabilized expression values. These metrics were plotted across differentiation time for each patient-derived line, with smoothed trends used to visualize average trajectories. To assess differences in epigenetic regulatory programs, a curated set of epigenetic regulator genes was examined. Mean expression values were calculated for iPSC-derived stromal samples and the Gutierrez eutopic cohort, and differences in mean expression (Δ expression = mean iPSC − mean Gutierrez) were visualized using bar plots.

### Statistical analysis

All statistical analyses were performed in R. Differential expression analyses were conducted using the DESeq2 framework with built-in normalization and dispersion estimation. Trajectory-based and pathway enrichment analyses incorporated appropriate ranking statistics and multiple-testing correction. For gene set enrichment and pathway-level analyses, FDR correction was applied using the Benjamini-Hochberg procedure. Unless otherwise stated, statistical significance was defined as FDR < 0.05. No overrepresentation analysis based on thresholded gene lists was performed; all pathway analyses were conducted using rank-based GSEA or per-sample ssGSEA approaches.
