# Supplementary material for: Endometriosis-derived iPSCs reveal conserved stromal maturation and endocrine responsiveness
Source: Sci Adv. 2026 Aug 19;12(34):eaeg2362. doi: 10.1126/sciadv.aeg2362 (PMC13488922; doi:10.1126/sciadv.aeg2362)
Supplement: Supplementary file 1 [file sciadv.aeg2362_sm.pdf]

Science Advances
AAAS

Supplementary Materials for

Endometriosis-derived iPSCs reveal conserved stromal maturation and endocrine responsiveness

Hannah McDowell *et al.*

Corresponding author: J. Julie Kim, j-kim4@northwestern.edu

Sci. Adv. **12**, eaeg2362 (2026)
DOI: 10.1126/sciadv.aeg2362

This PDF file includes:

Table S1

Supplemental Table 1: Patient information for iPSCs reprogrammed from PBMCs

Sample ID	Age	Diagnosis	Stage	LMP from blood draw	Race	Hormone Treatment	Used
PT 2	27	Endometriosis	2	3 months	White	cholecalciferol	RNA-seq
PT 4	39	Endometriosis	1	Unknown	White	Lupron	RNA-seq
PT 5	37	Endometriosis	1	6 weeks	American Indian or Alaska Native	norethindrone	RNA-seq
PT 6	37	Endometriosis	4	menses	White, Latin/Hispanic	norethindrone	RNA-seq
PT 9	34	Endometriosis	4	3 months	White	levothyroxine	RNA-seq
PT 10	32	Endometriosis	4	4 months	unknown	none	RNA-seq
PT 11	43	Endometriosis	1	7 weeks	White	TSH, cholecalciferol	RNA-seq
SSL008	27	No Endometriosis	NA	regular cycle	White	none	CM-THP1
SSL012	30	No Endometriosis	NA	regular cycle	White,	none	CM-THP1
SSL014	35	No Endometriosis	NA	regular cycle	White	none	CM-THP1
PT735	32	Endometriosis	3/4	NA	White	none	CM-THP1
PT774	40	Adenomyosis	NA	NA	Other	none	CM-THP1
